# The Endothelium in Acromegaly

**DOI:** 10.3389/fendo.2019.00437

**Published:** 2019-07-24

**Authors:** Pietro Maffei, Francesca Dassie, Alexandra Wennberg, Matteo Parolin, Roberto Vettor

**Affiliations:** ^1^Clinica Medica 3, Department of Medicine (DIMED), Padua University Hospital, Padua, Italy; ^2^Clinica Neurologica, Department of Neurosciences (DNS), Padua University Hospital, Padua, Italy

**Keywords:** IGF-1, endothelial cells, atherosclerosis, microcirculation, aneurysms, IMT, FMD, stiffness

## Abstract

Growth hormone (GH) and insulin like growth factor-1 (IGF-1) excess induce well-known deleterious effects on the cardiovascular system, especially after long-term exposition. Acromegaly, a condition of chronic GH and IGF-1 hypersecretion, is frequently associated to cardiovascular complications, although recent studies have shown a reduction in the prevalence of these comorbidities in well-controlled patients and a mortality risk similar to normal aging population. Many factors could contribute to the increased cardiovascular risk of acromegaly patients. Among these factors, the endothelium plays a key role in the pathogenesis of atherosclerotic plaques and could be considered an early marker of atherosclerosis and cardiovascular dysfunction. In this review we examined the relationship between GH/IGF-1 excess and the endothelium, from basic studies to clinical evidence. Many studies involving various arterial districts (microvascular arteries of retina, kidney and brain, and major vessels as carotid and aorta) showed that GH/IGF-1 excess promotes endothelial dysfunction via several different mechanisms. Increased endothelial proliferation, dysfunction of endothelial progenitor cells, increased oxidative stress, and compromised oxidative defenses are the main factors that are associated with endothelial dysfunction. In the general population, these alterations are associated with the development of atherosclerosis with an increased incidence of coronary artery disease and cerebrovascular complications. However, in acromegaly this is still a debated issue, despite the presence of many pro-atherogenic factors and comorbidities, such as hypertension, diabetes, sleep apnoea, and metabolic syndrome. Preclinical markers of atherosclerosis as arterial intima media thickness, pulse wave velocity and flow mediated dilation seem to be impaired in acromegaly and partly mediated by the endothelium dysfunction. In conclusion, the pathophysiology of endothelial dysfunction in the condition of GH and IGF-1 excess remains a crucial area of investigation to fully dissect the association of acromegaly with cardiovascular disease complications.

## Introduction

Endothelial cells are specialized components of the blood vessels which form a thin monolayer in the inner vessel wall called the endothelium. Abnormalities in endothelium physiology have been reported in a wide range of disorders including cardiovascular diseases, inflammation, tumor growth, and metastasis. It is well-known that the endothelium plays a key role in the pathogenesis of atherosclerotic plaques and, nowadays, endothelial dysfunction could be considered an early marker of atherosclerosis. In fact, an intact endothelium decreases the vascular tone, inhibits platelet adhesion, decreases the activation of the coagulation system, stimulates fibrinolysis, and inhibits the adhesion and migration of inflammatory cells (1).

Epidemiological studies have shown that insulin resistance and type 2 diabetes, hypertension, and dyslipidemia are frequent complications of acromegaly (2). All of these disorders have been associated with endothelial dysfunction and atherosclerosis in the general population (1). For this reason, the relationship between GH and IGF-1, the endothelium, and atherosclerosis have been widely investigated both in GH deficiency models, as well as in the opposed condition of GH excess, reporting in some cases discordant results. The aim of this review is to summarize the studies available on the effects of excess GH and IGF-1 on the endothelium, considering both basic and experimental research models, in particular in the context of acromegaly. Although the studies of the last decade have shown a decreased mortality rate in acromegaly, we believe is important to review this topic, because cardiovascular complications remain among the main cause of death in acromegaly, especially after long-term exposition to high levels of GH and IGF-1 (2–4).

## Study Identification

We conducted a systematic review of the literature searching the Medline/PubMed, ISI-Web of Knowledge, and Google scholar databases from January 1st, 1980 to December 31st, 2018. The keywords “acromegaly,” “endothelium,” “cardiovascular,” “stiffness,” “intimal medial thickness,” “IMT,” “flow mediated dilatation,” “FMD,” “aneurisms,” “microcirculation,” “atherosclerosis,” “endothelial cell,” “Growth hormone,” “GH,” “insulin-like growth factor 1,” “IGF-1” were used in various combinations. The search was extended to reference lists of relevant reviews. We excluded duplicate studies. We included prospective, cross-sectional, meta-analysis, review articles, and basic studies meeting the following criteria: written in English and inherent to the discussed matter. Studies were included regardless of their publication status or size. Studies not meeting these criteria were excluded.

## *In vitro* Basic Models of Excess GH and IGF-1

Chronically elevated levels of GH and IGF-1 have been associated with the development of cardiovascular complications, and their effects on vascular endothelium and vascular smooth muscle cells have been largely investigated *in vitro*.

*In vitro* studies have clearly shown that GH could act as an angiogenic factor by inducing endothelial cell proliferation, migration, and the formation of well-structured capillary-like tubes (5). These effects might also be explained by the ability of GH to modulate the cell cytoskeleton. In addition, GH exposition has been shown to enhance the proliferation of both human retinal microvascular endothelial cells (6) and brain endothelial cells (7). Human retinal microvascular endothelial cells respond to physiologic concentrations of hGH *in vitro* with enhanced proliferation (8, 9).

It has been reported that bovine aortic endothelial cells and rat aortic smooth muscle cells may secrete IGF-1 (10). In addition, human, bovine, and rodent endothelial cells produce large quantities of IGF binding proteins (IGFBP) *in vitro* (11). Hyperinsulinemia, in combination with IGF-1 elevation, could accelerate the proliferation of vascular smooth muscle cells and aggravate the ongoing process of atherosclerosis. King et al. (12) reported the presence of specific receptors for IGF-1 and IGF-2 on bovine retinal capillary and aortic endothelial cells. In particular, they discovered that retinal capillary endothelial cells exhibit a significant response to IGFs and insulin, suggesting that their elevation could accelerate the proliferation of retinal capillaries. Regarding macroangiopathy, they noticed that only the aortic smooth muscle cells, and not the endothelial cells, were responsive to insulin and IGFs. Unlike insulin and IGFs, GH did not have direct effects on the endothelium, suggesting that its possible pathophysiological actions on the vascular cells could be mediated through the IGF system. A few years later Bar et al. (13) reached the same conclusion studying *in vitro* the function of endothelial cells cultured from three different tissues: bovine adipose tissue microvessels, pulmonary arteries, and aorta. These authors observed that insulin, IGF-1, and IGF-2 stimulated the glucose and amino acid uptake and thymidine incorporation into DNA in the microvessel endothelial cells, but had no effect on the larger-vessel endothelial cells. Wilson et al. examined the effect of IGF-1 on human retinal endothelial cell survival following high glucose exposure and serum starvation (8). IGF-1 protected human retinal endothelial cells from high glucose-induced apoptosis and serum starvation. The authors suggested that IGF-1 was critical for human retinal endothelial cell survival, and that somatostatin analogs acting through the type 3 receptor could have direct effects on retinal endothelial cells. In addition, Spoerri et al. showed that cultured human retinal endothelial cells expressed IGFBP-3, while the exogenous administration of IGFBP-3 induced cell growth inhibition and apoptosis (9). Furthermore, SSTR agonists mediated the IGFBP-3 growth-inhibitory effects. Cercek et al. (14) confirmed the role of IGF-1 as an important autocrine or paracrine regulator of smooth muscle cell proliferation, and suggested that it may be significant in determining the cellular response in rat aortas after balloon denudation.

Several other tissues seem to be sensitive to the effects of IGF-1 on the endothelium. IGF-1 induced growth and chemotactic responses in bone endothelium, acting through the type I IGF receptor (15). Furthermore, IGFBP-3 has an autocrine/paracrine role in regulating various cell types in the primate corpus luteum, including endothelial cells (16). In contrast, proliferation of human umbilical vein endothelial cells was not significantly influenced by GH exposition. Endothelial cell migration and the underlying molecular mechanisms were explored using a human umbilical cord endothelial cell line (17). Treatment of these cells with IGF-1 but not GH stimulated the cell migration.

## Animal Models of Excess GH and IGF-1

Also the animal models of excess GH have shown the importance of GH and IGF-1 in the pathophysiology of endothelial and vascular function, dissecting many aspects of the complex regulatory pathway of endothelium involvement. Dilley and Schwartz studied a transgenic mouse model of the growth hormone gene C57/B16, with these mice overexpressing rat GH or bovine GH (bGH) (18). Unexpectedly, they found that arterial blood pressure was not elevated in mice, although there were significant increases in vascular wall mass with an average lumen diameter enlarged in several vascular sections of mesenteric blood vessels. Bohlooly et al. (19) studied the vascular function and blood pressure in a model of bGH transgenic mice. Hemodynamic studies of maximally dilated hindquarters of female bGH transgenic mice showed a higher resistance and a narrower average lumen diameter in comparison to control mice. In addition, a decreased sensitivity of vascular bed to the vasoconstrictor action of norepinephrine was suggested. Moreover, mean arterial blood pressure was increased and it was not salt sensitive. The reactivity to nitric oxide (NO) was similar in bGH transgenic and control mice, thus suggesting intact endothelial function (the importance of NO is described in following paragraphs). Further studies by the same group found that the carotid arteries from bGH transgenic mice had an impaired Ach-induced relaxation response (20). The carotid artery was affected earlier than the aorta, thus suggesting a vessel and time-specific deterioration of endothelial function in bGH transgenic mouse models. The authors speculate that endothelial dysfunction in bGH transgenic mice could be caused by increased oxidative stress or decreased oxidative defense. A different group of investigators recently showed, in bGH transgenic mice, that increased systolic blood pressure was associated with significant reduction of angiotensin-converting enzyme 2 and endothelial NO synthase expression in the kidneys (21). Furthermore, blood pressure elevation was dependent on age but independent of insulin resistance. Izzard et al. (22) studied a different transgenic mouse model of acromegaly, the giant bGH mice. The authors showed that the heart was hypertrophied and distal mesenteric arteries had significantly increased wall thickness and cross-sectional area, both of which were correlated with mouse body weight. However, the lumen diameter of mesenteric arteries and vascular contractility were not significantly different in comparison to controls. The authors speculated that in giant bGH mice, increased risk of mortality might be due to the development of hypertrophic cardiomyopathy, rather than atherosclerosis. Further investigations by Nishizawa et al. (23) showed that several indexes of oxidative stress of GH-transgenic rats, particularly in the left ventricular myocardium and vascular smooth muscle cell layer of the aorta, were increased. In particular, IGF-1 but not GH, induced reactive oxygen species (ROS) production in C2C12 myocytes.

## The Role of Endothelial Progenitor Cells (EPC), Nitric Oxide (NO), Hexarelin, Ghrelin and Other Hormonal Axis in GH-Mediated Endothelial Pathophysiology

The risk of atherosclerosis in the general population has been associated with low numbers or dysfunction of Endothelial progenitors cells (EPC). Thum et al. (24) demonstrated that exogenous IGF-1, but not GH, was an important regulator of EPC in mice and human subjects by increasing the EPC number, improving their colony forming and migratory capacity, and preventing EPC senescence. In addition, 1 year of rhGH (recombinant human Growth Hormone) replacement in adults with growth hormone deficiency ameliorated endothelial dysfunction and increased the number of circulating CD34+ cells (25).

NO is the primary mediator of endothelium-dependent relaxation. Many hormones, including steroids, insulin, and GH may modulate the production of endothelial nitric oxide synthase (eNOS) (26). Endothelium-derived NO is an important vasodilator, inhibitor of platelet adhesion and aggregation, inhibitor of monocyte adhesion, and inhibitor of vascular smooth muscle cell growth. Both *in vitro* and *in vivo* studies indicated that eNOS is activated though the GH/IGF pathway thus accounting for some of the effects of excess GH on endothelial dysfunction. Walsh et al. (27) studied the effects of exposure to IGF-1 on contractile responses of endothelium-intact rat tail artery. They observed that diminution in contractility was a direct effect of IGF-1 on the vasculature, probably mediated in large part by the release of NO. In addition, IGF-1 administration caused vasodilatation in experimental animals, which was blocked by inhibitors of eNOS (28). The IGF actions were also tested on aortic rings of spontaneously hypertensive and normotensive rats (29). Vasodilation evoked by IGF-1 was impaired in hypertensive rats, and after the removal of the endothelium or the inhibition of endothelial NO synthase, the vasodilation was blunted in both rat strains. Treatment of cultured human endothelial cells with GH resulted in significant increases of eNOS gene and protein expression, as well as NO release, whereas the production of intracellular reactive oxygen species was significantly reduced (30). Rapid formation of NO was detected in cultured endothelial cells exposed to IGF-1. Activation of the type I IGF receptor increased eNOS phosphorylation (31). Although a direct effect of GH on eNOS activity has yet to be proven, Gonzalez et al. (32) showed that GH induced NO-mediated vasodilatation in isolated aortic rat rings, in a cumulative dose-dependent manner through the interaction with the GH receptor. In Ames dwarf aortas, a model of GH deficiency, there was a less abundant expression of eNOS and the acetylcholine-induced relaxation was also decreased. Finally, in cultured wild-type mouse aortas and in human coronary arterial endothelial cells, treatment with GH and IGF upregulated the expression of eNOS (33).

Hexarelin and ghrelin are two potent GH secretagogues that have many effects on the cardiovascular system. In particular, ghrelin has been shown to increase myocardial contractility and cardiac output, repair endothelial cells, promote vascular endothelial function, and inhibit vascular inflammation, vascular smooth muscle cell proliferation and platelet aggregation. In aortic rings of the hypophysectomized rats it has been shown that GH and hexarelin treatment prevent endothelial vasodilator dysfunction (34). In cultures of rat endothelial cells derived from brain microvessels, ghrelin exerted a marked *in vitro* antiangiogenic action, and the mechanism underlying this effect was shown to involve the inhibition of TK/MAPK-dependent cascades (35). Shinde et al. suggested that NO synthase inhibition exaggerates the hypotensive response to ghrelin (36). The Ca-activated, K-channel-mediated, ghrelin-evoked decrease of mean arterial pressure may be significant in states of endothelial dysfunction associated with reduced NO availability. Ghrelin has a protective receptor-dependent effect in the porcine coronary artery by blocking homocysteine-induced endothelial dysfunction, improving eNOS expression, and reducing oxidative stress (37). Ghrelin stimulates cardiac microvascular endothelial cell angiogenesis through GH secretagogue receptor mediated MEK/ERK and PI3K/Akt signal pathways, indicating that the two pathways are required for full angiogenic activity of ghrelin (38). Ghrelin treatment markedly improved limb perfusion by promoting the generation of new capillaries and arterioles within the ischemic hind limb via endothelium-dependent vasodilatory responses to acetylcholine. Molecular analysis revealed that ghrelin's angiogenic properties were associated with activation of pro-survival Akt/vascular-endothelial growth factor/Bcl-2 signaling cascade, thus reducing cell death and subsequent fibrosis (39). Furthermore, it has been shown that acylated ghrelin protects aorta damage post-myocardial infarction via activation of eNOS (40).

Kulungowski et al. studied the expression of androgen, estrogen, progesterone, and GH receptors in vascular malformations (41). In particular, GH receptor expression was found to be significantly increased in arteriovenous, lymphatic, and venous malformation tissues compared with controls. The GH receptor was present primarily in the endothelium/perivasculature malformations, whereas in normal tissues the GH receptor was located only in the stroma. Age, sex, and location did not influence GH receptor expression.

These studies confirm the key role of GH and IGF-1 as fundamental actors in the complex network that regulates endothelial functions. The final and precise pathophysiology is far from being fully understood. At this moment many aspects remain obscure and this could be the one the reason of some apparent contradiction in clinical studies that have been discussed below.

## Atherosclerosis in Acromegaly

In acromegaly patients, the incidence of endothelial dysfunction and its subsequent evolution toward atherosclerotic disease are still a matter of debate. The prevalence of atherosclerosis in acromegaly is still unknown. In fact, some studies reported an increased carotid intimal media thickness (IMT) while other authors have shown a prevalence of artery disease, which was comparable to that of general population (42–44).

### Pathogenesis of Atherosclerosis

As previously stated, GH plays a crucial role in the endothelium—it reduces vascular inflammation, stimulates EPC, improves fibrinolysis, and modulates the expression of adhesion molecules (45). On the other hand, IGF-1 may play a dual role in the origin of atherosclerotic plaques: (1) pro-atherogenic, promoting vascular smooth muscle cell proliferation and eventual migration and activation of macrophages; and (2) protective, promoting plaque stability, smooth muscle cell survival, and endothelial repair through the EPC (46).

In acromegaly patients, both excess GH and IGF-1 promote endothelial dysfunction and atherosclerosis through many different mechanisms:

- favoring cardiovascular comorbidities such as hypertension, insulin resistance, diabetes, sleep apnea, lipid abnormalities, metabolic syndrome, and obesity;
- favoring endocrinological comorbidities, such as sex hormone imbalance;
- increasing oxidative stress;
- increasing pro-inflammatory cytokines;
- inducing the modification of hemodynamic forces;
- modifying endothelial repair;
- inducing expression of adhesion molecules; and
- inducing morphological vascular alterations through the proliferation of vascular smooth muscle cells (47).

### Oxidative Stress

Oxidative stress is characterized by increased formation of ROS. ROS induce a reduced antioxidative capacity and a decreased availability of NO, which is important for vascular homeostasis. NO acts on smooth muscle cells, circulating blood platelets and leukocytes, and it can oxidize low density lipoproteins (OxLDL). OxLDL cause promotion of foam cell formation, chemotaxis, activation of leucocytes, stimulation of monocytes, neutrophil adhesion to endothelial cells, and impairment of endothelium-derived NO release of endothelin-1 endothelial secretion. OxLDL are associated with preclinical atherosclerosis and they could be considered a negative prognostic factor for cardiovascular outcomes (48).

In acromegaly, some studies have demonstrated the presence of higher LDL oxidation and lipid peroxidation; although other recent studies have not shown higher oxidative stress. Boero et al. studied 15 patients with active acromegaly and these authors demonstrated significantly higher OxLDL in patients with acromegaly in comparison to controls (49). Furthermore, increased oxidative stress in acromegaly patients persisted after adjusting the OxLDL for glucose and insulin levels. The authors also showed a qualitative change of the lipoprotein profile and high cholesteryl ester transfer protein activity, which was able to increase both LDL triglyceride content and Lp(a) number. Also, ceruloplasmin (CP), MPO, 15-lipoxygenase, and inducible NO synthase have been found in animal and human atherosclerotic lesions, and could cause or contribute to LDL oxidation. The authors found that only CP activity was increased, and this could be an important risk factor for cardiovascular disease in acromegaly. In this study, oxidative stress was increased, but there were no differences in antioxidant potential between acromegaly patients and controls (49). Additionally, Yarman et al. found higher oxidative stress and thiobarbituric acid reactive substance levels (TBARS), which reflects the plasma concentrations of lipid peroxides, in newly diagnosed acromegaly patients. The authors showed decreased lipid peroxide levels after acute Octreotide administration (50). Anagnostis et al. studied 15 patients with acromegaly and demonstrated higher oxidative stress in comparison to controls, and they showed a reduction of catalase activity, glutatione concentration, total oxidized glutathione, NO levels, and higher TBARS (51). Moreover, among 51 acromegaly patients, Ilhan et al. showed a reduction of superoxide dismutase and total antioxidant capacity levels, compared to controls, while acromegaly treatment did not substantially improve these findings (52).

We must point out that recent studies do not seem to support the theory of increased oxidative stress occurrence in acromegaly. Ozkan et al. showed an increased carotid IMT, but there were no differences in molecule high mobility group box 1, which is essential for DNA repair, or OxLDL, between acromegaly patients and controls. These findings suggest that early atherosclerosis in acromegaly was not sustained by inflammation or oxidative stress (53). Finally, Kirilov et al. showed increased levels of endothelin 1 in patients with active disease compared to cured acromegaly patients; however, homocysteine levels did not differ between acromegaly patients and controls (54).

### Lipid Disorders

The effect of acromegaly and its treatment on plasma lipids and lipoprotein levels has been summarized in Table 1 (55).

**Table 1:** Lipid and lipoprotein levels on acromegaly.

	**Acromegaly**	**Acromegaly after treatment**
Total Cholesterol	Variable	Variable
LDL Cholesterol	Variable	Decrease
HDL Cholesterol	Decrease	Increase
Triglycerides	Increase	Decrease
Lp(a)	Increase	Decrease
Lipoprotein associated phospholipase A2	Unchanged	

The elevation of plasma triglycerides in acromegaly is usually associated with a combination of increased production rate and a decreased clearance (56–58); insulin resistance and hyperglycemia could contribute to the abnormalities in triglyceride metabolism in acromegaly. A recent meta-analysis of 13 studies on 305 patients showed a mean reduction of plasma triglycerides after somatostatin analog treatment in acromegaly (59). Concerning HDL decrease, many studies have reported a reduction of lecithin cholesterol acyltransferase, cholesterylester transfer protein, hepatic lipase, and phospholipid transfert protein, which are all involved in lipid metabolism (60, 61). Some studies have shown increased Lp(a) levels in patients with acromegaly (62). In particular, Arosio et al. showed that this subfraction distribution remained unmodified during prolonged Octreotide therapy among 20 patients with acromegaly (63).

### Pro-inflammatory Citokines

Several studies investigated the levels of pro-inflammatory cytokines in acromegaly patients. Indeed, endothelial cells play an active role in acute and chronic inflammatory responses with an activation program that confers a “pro-inflammatory endothelial phenotype.” This pattern is characterized by secretion of IL-1, TNF-α, INF-gamma, IL-8, MCP-1, IL-6, CRP, IL-11, GM-CSF, YKL-40 (human cartilage glycoprotein 39—a biomarker of acute and chronic inflammation made by endothelial cells, smooth muscle cells, macrophages, and neutrophils), and procalcitonin. Also, angiogenic factors, such as the vascular endothelial growth factors (VEGF), angiopoietin-2 hepatocyte growth factor (HGF), endostatin, and angiogein contribute to the maintenance of endothelium layer integrity. Further, the exposure of subendothelial tissue can promote endothelial dysfunction and atherosclerosis. Another important mechanism involved in endothelial dysfunction is the capacity of dysfunctional adipocytes to generate pro-inflammatory adipokines, such as adiponectin and leptin, which can activate macrophages, the microvascular endothelium, and NFKB dependent pathways (64).

Results of studies on pro-inflammatory cytokines in acromegaly have been summarized in Table 2. Interestingly, CRP has been found to be reduced in active acromegaly despite the high risk of cardiovascular outcomes among acromegaly patients (65).

**Table 2:** Pro-inflammatory cytokines in acromegaly.

**References**	**Population**	**Results**
Arikan et al. (66)	22 naïve patients 26 controls	↑ TNF-α, IL-8 = hsCRP, omocysteine, IL-1β, IL-2R, IL-6, IL-10
Wolters et al. (67)	12 patients 24 controls	↑ IL-6
Ueland et al. (68)	47 naïve patients 25 controls	↑ IL-1β, OCN, ucOCN = adiponectin, RBP4, IL-6 ↓ leptin, IL-1Ra
Andreassen et al. (69)	21 patients 42 controls for CRP 63 controls for YKL-40 63 controls for IL-6	= IL-6 ↓ CRP, YKL-40
Silha et al. (70)	35 active patients untreated 101 controls	↑ angiogein, VEGF only in male = HGF, endostatin, VEGF, angiopoietin, VEGF receptor
Ozkan et al. (53)	39 patients 40 controls	↓ CRP
Ozkan et al. (71)	33 active patients 22 non-active patients 20 controls	= procalcitonin (correlation with IMT), CRP,
Kaluzny et al. (72)	77 patients 56 active 21 controls	↓↓ in active patients ↓ in controlled patients
Potter et al. (73)	12 active patients 12 controlled patients 12 controls	= CRP
Vilar et al. (74)	62 patients 50 active patients 12 controls patients 36 controls	= hsCRP
Nagai et al. (75)	13 patients 16 controls	= VEGF
Sesmilo et al. (76)	48 patients 47 controls	↓ CRP = IL-6, omocystein

### Cell Adhesion Molecules

Endothelial dysfunction is also characterized by overexpression of cell adhesion molecules that could be released into the systemic circulation; these molecules have inflammatory properties, mediate the adhesion of leucocytes, and have a relevant role in the progression of atherosclerosis (77).

Accordingly, Topaloglu et al. showed higher serum levels of intercellular adhesion molecule 1 (ICAM-1) and vascular cell adhesion molecule 1 (VCAM-1) in patients with acromegaly compared to controls. Furthermore, E selectin levels were found to be significantly higher in patients with active acromegaly disease, while there were no differences of these markers between active acromegaly patients and cured ones. The Hs-CRP, homocysteine, and lipid profiles were not different between the three groups. Serum VCAM-1 levels were significantly higher in hypertensive patients than in normotensive patients. ICAM was higher in patients without diabetes compared to controls (77). Also, in this case, not all studies support a significant alteration of the adhesion molecules in acromegaly. According to Boero et al. VCAM-1 levels are not elevated in acromegaly (78).

### Pro-thrombotic Mediators

Acromegaly is associated with a possible mild hypercoagulability state, which is of uncertain clinical relevance. In fact, some studies found increased levels of fibrinogen and plasminogen activator inhinibitor-1 (PAI-1), and decreased levels of tissue plasminogen activator (t-PA) and tissue factor pathway inhibitor (TFPI) in acromegaly. Tissue factor (TF), PAI-1, and Von Willebrand factor (vWF) are pro-thrombotic mediators, which have been involved in endothelial activation and atherosclerosis pathogenesis. These data in acromegaly patients suggest an increased fibrinolysis with thrombogenic potential, and these findings seem to be partially reversible after the treatment of acromegaly (79).

### Endothelial Repair

The EPC derive from bone marrow and/or the vascular wall. They contribute to neovascularization in response to ischemia, and their levels are related to cardiovascular disease outcome. EPC promote endothelial repair against endothelial dysfunction. EPC reduction could be considered a risk factor for atherosclerosis (80).

In the literature, there are contrasting results on EPC levels in acromegaly. In an early study among 55 patients with acromegaly, it was found that acromegaly patients, as compared to controls, showed higher EPC levels, which positively correlated to IGF-1 (81). On the contrary, another study showed a significant reduction of EPC levels in acromegaly patients compared with controls. In this study, EPC levels correlated with IGF-1, fasting plasma glucose, and homeostasis model assessment index of insulin resistance. The authors showed a significant decrease of EPC levels in acromegaly patients after somatostatin analog treatment (82). Fadini et al. showed a reduction in circulating myeloid calcifying cells (MCCs) in 44 patients with acromegaly. The MCCs levels remained low even years after the cure of acromegaly. MCCs are usually increased in patients with high cardiovascular risk, such as diabetic patients. They promote ectopic calcification, and in ApoE^−/−^ atherosclerotic-prone mice they increase plaque calcification (83).

## Clinical Manifestation of Atherosclerosis in Acromegaly

### Preclinical Markers of Endothelial Dysfunction

Among acromegaly patients, vascular abnormalities have been widely described. As far as preclinical markers of endothelial dysfunction are considered, there are structural abnormalities, such as impaired intima media thickness (IMT), and functional abnormalities, such as impaired flow-mediated dilation (FMD) or arterial stiffness.

Carotid IMT could be measured using ultrasonography, and is associated with an increase of cardiovascular risk in the general population. In particular, the relative risk of stroke or myocardial infarction is associated with IMT augmentation (84, 85). In acromegaly, many authors studied the IMT of carotid arteries reporting discordant results. Colao et al. (86) reported significant augmentation of IMT in acromegaly patients in comparison to controls. Furthermore, 6 months of treatment with Lanreotide did not produce significant IMT reduction. Cansu et al. (87) confirmed higher IMT in a group of acromegaly patients; however, the IMT in controlled acromegaly patients was not significantly different from uncontrolled patients. Lin et al. (88) found that the IMT in controlled acromegaly patients was significantly lower than the IMT of active acromegaly patients. On the other hand, Otzuki et al. reported no significant difference of IMT between acromegaly patients and controls (89). In addition, Topaloglu et al. reported a slight inferior IMT in active acromegaly patients in comparison to controlled acromegaly patients; however, this difference was not statistically significant (77).

Another non-invasive functional vascular test that could predict the development of atherosclerosis is the measurement of FMD. Endothelial dysfunction can be studied with ultrasonography by measuring the post-ischemic FMD of conduit arteries. Impaired FMD is an early marker of atherosclerosis and is associated with cardiovascular events (90–93). Studies of FMD in acromegaly are concordant in reporting lower FMD in acromegaly patients in comparison to controls (69, 94). When comparing active acromegaly patients and controlled acromegaly patients, discordant results are reported by some authors. Brevetti et al. (42) reported significantly lower FMD in 18 active patients, in comparison to 12 controlled acromegaly patients, while Ozkan et al. (53) paradoxically found a slightly non-significant lower FMD in controlled patients in comparison to active acromegaly patients.

Arterial stiffness could be measured using several different non-invasive methods. The gold standard method is the aortic pulse wave velocity (aPWV), which analyzes arterial ability to expand and contract with cardiac pulsation and relaxation. PWV is correlated with cardiovascular disease in the general population (95–97). PWV can also be measured in other anatomic districts, such as the carotid artery (98). Other methods to study arterial stiffness are the aortic stiffness index (ASI), otherwise known as the beta index, which can also be indirectly derived from PWV by an equation (99), and the augmentation index (AI), a central hemodynamic index that predict cardiovascular events (100). Arterial stiffness is influenced by reversible and irreversible changes. Reversible features include the vascular wall tone and endothelial function modulated by mediators, such as NO, angiotensin II, and endothelin-1. Irreversible changes include the vascular wall alterations due to deposition of extracellular matrix components like collagen and elastin. Excess GH/IGF-1 in acromegaly may involve all these abnormalities (101, 102). Cansu et al. (87) found a higher PWV in acromegaly patients in comparison to controls, but did not find a significant difference between active and controlled acromegaly patients. Găloiu et al. (103), analyzed carotid wall parameters of acromegaly patients in comparison to controls and found that carotid PWV, AI, and the β index were significantly lower in active acromegaly patients in comparison to controls. Contrastingly, Yaron et al. found no differences of aPWV between acromegaly patients and controls, including active vs. controlled acromegaly patients (104).

As aforementioned, some studies did not find differences in these parameters between active and controlled acromegaly patients, suggesting that the negative effect of excess GH and IGF-1 may persist despite disease control. We recently performed a meta-analysis of 27 studies that reported consistent data about IMT, FMD, and PWV in acromegaly. We found that IMT was significantly higher in a global population of 474 acromegaly patients in comparison to 373 controls. Moreover, IMT remained significantly higher in 322 active acromegaly patients in comparison to 245 controlled acromegaly patients. Also, FMD was significantly impaired in acromegaly in comparison to controls; the result was significant also for active acromegaly patients in comparison to controlled acromegaly patients. Finally, PWV was significantly higher in a global population of 239 acromegaly patients in comparison to controls. In the meta-regression analysis, we found that the disease activity had a more evident effect on IMT impairment in the population with a lower percentage of hypertensive or diabetic acromegaly patients (105).

The treatment of acromegaly seems to have a positive effect on ameliorating these preclinical markers, but no definite results emerge from the literature. Sakai et al. found that FMD significantly improved in acromegaly patients after trans-sphenoidal surgery, while the IMT and PWV did not show significant changes (106). Colao et al. (107) studied 25 naïve acromegaly patients before and after 6-month treatment with octreotide LAR, reporting a significant reduction of IMT. De Martino et al. studied 10 patients with acromegaly before and after 18-month treatment with Pegvisomant and found a significant decrease of FMD, but no significant differences in IMT (108). As we stated in the meta-analysis, we think that further prospective studies are necessary to determine variations of these preclinical parameters after the normalization of excess GH and IGF-1, and to identify their precise role in development of atherosclerosis and cardiovascular risk.

### Microcirculation

In addition to the studies regarding macrovessels, the alteration of microvessels are another field of interest in order to assess the cardiovascular risk in acromegaly. The study of microcirculation involves the ability to visualize microvessels using optical magnification in the range of 10-700X with a microscope. Microcirculation consists of arterioles, capillaries, and venules (109). Microcirculation has been widely studied for the prediction of cardiovascular events (110–112). Small arteries and arterioles are the principal determinant of peripheral resistance and hypertension pathogenesis (113, 114). Structural alterations of microvessels, such as increased wall thickness, are predictors of cardiovascular disease in hypertensive subjects. In particular, increased wall-to-lumen ratio was demonstrated to be a strong predictor of cardiovascular events in high-risk populations (115). In addition, cutaneous circulation resistance plays an important role on systemic vascular resistance (116).

In acromegaly, some studies on microvascular alterations have been conducted. Initial studies were based on autoptical series, and showed a proliferative wall thickening of intramural small vessels in the hearts of acromegaly patients (117). Chanson et al. analyzed the blood flow in the brachial artery, and found it was not significantly different between acromegaly patients and controls. However, the brachial artery mean blood flow and the mean blood velocity were lower in patients with higher vascular resistance in acromegaly patients vs. controls (118). Evans and Davies suggested that these functional microvascular abnormalities, and, in particular, the dysregulation of arterial tone and impairment of endothelial function might play a role in the arterial disease observed in acromegaly (119).

Schiavon et al. (120) studied the nailfold microcirculation bed by capillaroscopy, and found a reduced number and length of capillaries in acromegaly patients associated with a significant increase of tortuous capillaries. They found only limited differences between active and controlled acromegaly patients, concerning density and tortuosity of capillaries. In addition, they found that these alterations were independent of diabetes or hypertension, so they hypothesized an independent role of acromegaly on microcirculation anatomy. The mechanism behind these anatomical alterations remains completely unknown. Maison et al. (121) studied the cutaneous vasoreactivity responses at the palm and dorsum of the hands in active acromegaly patients compared to a group of matched controls. They studied the cutaneous circulation velocities after exposure to a temperature of 44°C to study vascular muscle function (122) after shear-stress to study NO-mediated vasodilatation (123), and after a sympathetic response induced by cold stress on the opposite hand (124). They found similar responses to the warm test in acromegaly patients and controls. The ischemic-response produced a vasodilatation in both groups, but the response indexes were significantly reduced by 50% in acromegaly patients in comparison to controls. In addition, the cold test showed a higher, but not significant, grade of vasoconstriction in acromegaly patients compared to controls. Abnormal microvascular blood flow was also found by Krsek et al. (125) comparing active acromegaly patients to healthy controls. They analyzed forearm microcirculation using laser Doppler fluximetry before and after post-occlusive reactive hyperemia or thermal hyperemia, finding a significant reduction of both these parameters in acromegaly. Interestingly, they did not find significant differences of IMT between acromegaly patients and controls; however, IMT negatively correlated with post-obstructive reactive hyperemia. These authors hypothesized an association of macrovascular and microvascular alterations in acromegaly. Rizzoni et al. compared three different groups of subjects (acromegaly patients, hypertensive patients, and normotensive healthy controls), and studied the structure of subcutaneous arterioles. They performed a biopsy of gluteal fat and found significant structural alteration of microvessels in acromegaly, showing a greater vessel diameter, media thickness, and wall thickness in comparison to hypertensive controls. In addition, they found a greater media-to-lumen ratio in acromegaly patients compared to controls. Hypertensive patients had a significantly higher media-to-lumen ratio and media thickness and a significantly smaller internal diameter compared to normotensive subjects. In contrast to hypertensive patients, who are characterized by eutrophic remodeling, acromegaly patients seem to have inward hypertrophic remodeling. They did not find significant differences between normotensive and hypertensive acromegaly patients. These alterations in acromegaly could be due to increased levels of IGF-1, since they observed a significant correlation between media-to-lumen ratio and serum IGF-1 (126).

Paisley et al. (127) also analyzed the vessels derived from gluteal fat biopsies of acromegaly patients, in comparison to a group of matched controls, by using pressure arteriography. They studied the capacity of vasoconstriction and vasodilatation of these vessels after the administration of adrenaline and acetylcholine or after the administration of NO synthase inhibitors. Within structural alteration, they found a significant impairment in wall-thickness, probably due to wall hypertrophy, and a small artery wall-to-lumen ratio and cross-sectional area in acromegaly patients in comparison to controls. These alterations significantly improved after treatment, although without complete normalization. Among functional features, they found no differences between acromegaly patients and controls in the response to adrenaline, but vasodilatation in response to acetylcholine was significantly lower in acromegaly, with a significant difference between active and controlled subjects.

From these results, we can state that alterations of microcirculation in acromegaly are far from being fully understood. In particular, the connection between microvascular impairment and cardiovascular risk and mortality remains unclear. Recently, Tellatin et al. (128) performed transthoracic Doppler echocardiography before and after administration of adenosine in 40 acromegaly patients to assess the coronary flow reserve (CFR). They demonstrated that CFR, a marker of coronary microvascular function, was lower in acromegaly patients in comparison to controls. Interestingly, they found an independent association between CFR and IGF-1, thus concluding that IGF-1 could have an important role in microvascular dysfunction in acromegaly that could be partially improved by disease control.

## Coronary Heart and Cerebrovascular Disease

As aforementioned, it seems that the preclinical factors that lead to endothelial, microvascular and macrovascular dysfunction are impaired in patients affected by acromegaly, but questions still remain: do these factors increase the risk of cardiac or cerebral damage in this population? Is the cardiovascular mortality increased due to these factors? Studies concerning cardiovascular and cerebrovascular damage and mortality are far from conclusive especially in recent years (2, 4).

### Coronary Heart Disease

The prevalence of atherosclerosis plaques in acromegaly is not well-established. Few studies have reported on the presence of carotid plaques and only some authors have reported prevalence of artery disease comparable to the general population (42–44). In the recent Liege acromegaly survey, the prevalence of clinical manifestation of atherosclerosis at diagnosis of acromegaly was in the range of 3.5% for ischemic heart disease and 3.0 % for myocardial infarction (129). Other European and Mexican population studies or registries have reported wide estimates of coronary disease ranging from 2.5 to 12% (130–133). A multicenter German study of 479 patients reported a standardized incidence ratio (SIR) of myocardial infarction in acromegaly that was similar to that of general population [0.89 (0.47–1.52), *p* = 0.80] (134). Also a Danish study demonstrated that myocardial infarction was not increased in patients with acromegaly compared to the general Danish population (135). Even post-mortem studies showed the occurrence of coronary artery disease only in patients with long-term acromegaly disease activity (136).

The evaluation of coronary artery calcium as assessed by heart-CT showed that 41% of acromegaly patients were at risk of coronary atherosclerosis independent of disease activity. Calcium deposits in the acromegaly heart may not be progressive, as they are in the general population, which would be consistent with the hypothesized protective effect of excess GH (137). In another prospective study, the risk of coronary artery disease was low and no acromegaly patient developed a major cardiac event during the period of study (138). CAD risk in newly diagnosed acromegaly patients was low and remained stable after successful treatment. Coronary artery calcium content was lower in acromegaly patients than in controls, suggesting that excess GH *per se* does not carry an additional CAD risk (139). Results from the studies on coronary artery calcium investigated by heart-CT have been summarized in Table 3.

**Table 3:** Studies on coronary artery calcium investigated by heart-CT in acromegaly.

	**References**	**Patients and methods**	**Time of follow-up**	**Risk factors**	**Outcome**	**Agaston score >0**
Increased coronary calcium	Cannavo et al. (136)	39 patients Cross-sectional	-	Diabetes Hypertension High Framingham risk score	Not known 1 patient with previous IM	56%
	Herrmann et al. (139)	30 patients Cross-sectional	-	High Framingham risk score Long disease duration	-	53%
	Ragonese et al. (140)	52 patients [39 patients from previous study Cannavo et al. (136)] Prospective	5 years		13 cardiovascular events with 5 deaths	31%
Not increased coronary calcium	Bogazzi et al. (137)	52 patients Prospective	5 years	High Framingham risk score	No cardiovascular events	36%
	Akutsu et al. (138)	25 patients at diagnosis of acromegaly	4.6 ± 1.1 years	Age, sex and ESC risk score	No cardiovascular events Agaston Score leels was stable during follow-up	20% lower than controls
	Dos Santos et al. (141)	56 patients Cross-sectional	-	High Framingham risk score Age Inverse correlation between Agaston left anterior descending coronary and IGF-1 Agaston DA e IGF-1	-	28% no differences with controls

### Cerebrovascular Disease

Cerebrovascular events are increased in acromegaly (142) and could significantly affect mortality. This was confirmed in a recent meta-analysis where the authors compared the SMR of 5 studies published before 2008 and 3 studies published after 2008: the SMR was 2.42 before 2008 and 3.38 after 2008 (4). The results from the German Acromegaly Registry (479 patients: 15 patients with stroke, standardized incidence ratio = 1.17 *p* = 0.61) and the Danish national study (hazard ratio 1.1) showed that stroke incidence did not differ from that of general population (133, 134). The incidence of stroke at first diagnosis in acromegaly patients in the Liege survey was 4.5%. Common risk factors for cerebrovascular events in acromegaly are hypertension, insulin resistance, diabetes mellitus, cardiomyopathy, arrhythmias, valvular heart disease, dyslipidemia, increased IMT, hypopituitarism, pituitary surgery complication (1,153 patients with all type of pituitary adenoma, stroke incidence 0.3%), and cranial radiotherapy (143).

Acromegaly patients and patients with other pituitary adenomas who underwent radiotherapy showed elevated incident stroke mortality rates, with a SMR of 4.42 (range 2.71–7.22) (144), nevertheless the direct link between RT and cerebrovascular disease is still unknown.

A recent cerebral MRI study was done comparing acromegaly patients treated with radiotherapy to a control group of acromegaly patients not treated with radiotherapy, matched for age, gender, clinical and adenoma features. The authors found that acromegaly patients treated with radiotherapy had significantly increased white matter signal abnormalities in the temporal lobe, basal ganglia, and infratentorial regions. One patient suffered from a stroke due to right internal carotid artery occlusion (145). The authors found a higher rate of white matter changes in acromegalic patients over a long-term follow-up after radiotherapy (>10 years) (146).

In the literature, there are no studies comparing the cerebrovascular damage of patients who underwent conventional radiotherapy to patients who had pituitary radiosurgery. Furthermore, there are no prospective studies comparing the two types of radiotherapy. The techniques of conventional radiotherapy have been improved in the last few years, and cerebrovascular disease and mortality have not been systematically studied after RT. In a meta-analysis, Abu Dabrh et al. concluded that radiosurgery produced better biochemical remission with a lower rate of hypopituitarism, but the evidence was not robust due to the non-comparative nature and the high heterogeneity of studies evaluated. The authors showed no results in cerebrovascular outcomes (147). Studies have shown that conventional radiotherapy increases risk of mortality (148, 149), and studies of FSRT reported an incidence of stroke between 0 and 9% (150). Among patients treated with radiosurgery, the authors found cerebrovascular events in 2 studies out of 35 (one case of coronary artery stenosis and two of transient ischemic attack) (151, 152). After conventional radiotherapy, the rate of cerebrovascular disease is influenced by radiation dose and atherogenesis due to radiotoxicity. Radiosurgery seems to cause less vascular disease, and modern radiotherapy techniques improve cerebrovascular mortality and morbidity risk (132, 153).

### Aneurysms

Among the possible vascular complications of acromegaly cerebral aneurysms also have to be considered (154). Some case reports described the presence of cerebral aneurysms in acromegaly, while only two studies analyzed cerebral arteries in a large cohort of patients (155–158). In the first study the authors examined 161 acromegaly patients with cerebral angiography MRI (MRA) or computed tomography angiography. Surprisingly, they found 40 newly diagnosed intracranial aneurysms in 26 patients (10 patients had multiple aneurysms) while 2 patients previously had subarachnoid hemorrhages due to aneurysm rupture. The incidence of aneurysms in this population was higher than of that in the general population analyzed with the same technique. Most of these findings were located in the intracranial tract of the internal carotid artery (67.5%), which is different from those of general population, thus suggesting a possible different pathogenesis of aneurysms in acromegaly (159). Another study investigated retrospectively the prevalence of cerebral aneurysms by MRA in 208 acromegaly patients, who were compared to a control group of 7,390 subjects who underwent a “brain check-up.” The prevalence of aneurysms in acromegaly patients was significantly increased (4.3%), with a higher prevalence in male subjects. The aneurysms were mostly found at the level of internal carotid artery, basilar artery, anterior cerebral artery, and middle cerebral artery. In addition, the authors identify acromegaly as a significant risk factor for aneurysm in multiple logistic regression models (160).

The presence of aneurysms had a positive correlation with GH values and poor disease control. In the literature reported risk factors for aneurysms pathogenesis in acromegaly include pituitary surgery, local radiotherapy, poor disease control, pituitary adenoma *per se*, vascular infiltration of adenoma, hypertension, diabetes, dyslipidemia, vascular effects of GH and IGF-1, such as proliferation of smooth muscle and endothelial disfunction, atherosclerosis, and altered turnover of collagen components (42, 161–164).

Aneurysms have also been sporadically reported in the ascending aorta (165) and femoral artery (166).

## Conclusions

Acromegaly is associated with increased mortality when not adequately treated. Although recent studies have shown that neoplasms are the main cause of death in acromegaly, the cardiovascular complications still represent a therapy challenge and a clinical burden. GH and IGF-1 excess exerts many actions on cardiovascular system and acromegaly is still associated with several risk factors that contribute to atherosclerosis as hypertension, diabetes and dyslipidemia. The specific mechanisms involved in the pathophysiology of cardiovascular comorbidities in acromegaly are not fully understood. The endothelium plays a key role in the pathogenesis of atherosclerotic plaques, and its derangement could be considered an early marker of atherosclerosis and cardiovascular dysfunction in the general population. In this review we examined the relationship between GH/IGF-1 excess and endothelium, from basic studies to clinical evidence. Basic studies have shown how the GH and IGF-1 are connected to a complex network of regulation of endothelial function, and it seems that many factors may contribute to a pro-atherogenic environment profile through the endothelium. Conversely, the studies involving pre-clinical and clinical markers of macro- and micro- vascular damage have contradictory results. Acromegaly patients do not always present with clear atherosclerotic damage and coronary heart disease in comparison to the normal population. Probably, the specific treatment at an early stage of disease of acromegaly and its systemic complications leads to important clinical and cardiovascular benefits. Further studies will clarify the effective role of endothelium involvement in acromegaly cardiovascular complications.

## Author Contributions

PM, FD, and MP conceived of and wrote the manuscript. AW did manuscript editing. PM and RV were in charge of overall direction and planning.

### Conflict of Interest Statement

The authors declare that the research was conducted in the absence of any commercial or financial relationships that could be construed as a potential conflict of interest.
